# Oxidized-Desialylated Low-Density Lipoprotein Inhibits the Antitumor Functions of Lymphokine Activated Killer Cells

**DOI:** 10.7150/jca.55526

**Published:** 2021-06-16

**Authors:** Jesús S Aguilar Díaz de león, Honor L Glenn, Mark Knappenberger, Chad R Borges

**Affiliations:** 1 School of Molecular Sciences and The Biodesign Institute - Center for Personalized Diagnostics, Arizona State University, P.O. Box 876401, Tempe, AZ 85287, USA.; 2 School of Life Sciences and The Biodesign Institute - Center for Immunotherapy, Vaccines and Virotherapy, Tempe, AZ 85287, USA.; 3 School of Life Sciences and The Biodesign Institute - Center for Personalized Diagnostics, Arizona State University, P.O. Box 876401, Tempe, AZ 85287, USA.

**Keywords:** lymphokine activated killer (LAK) cells, atherosclerosis, oxidized-desialylated low-density lipoprotein (LDL), cancer

## Abstract

Elevated concentrations of circulating low density lipoprotein (LDL) that is abnormally oxidized and desialylated is both a precursor to and a hallmark of atherosclerosis. Peripheral blood mononuclear cells (PBMCs) treated *in vitro* with interleukin-2 (IL-2) become lymphokine activated killer (LAK) cells, the primary effectors of which are NK cells and NKT cells. LAK cells display antitumor functions such as increased cytotoxicity and IFN-γ production, and they have been evaluated as a potential cancer therapeutic. Atherosclerotic processes may influence innate immunity against cancer. Because prior studies have shown that low density lipoprotein (LDL) reduces T-cell and NK cell antitumor functions, we asked whether oxidized-desialylated LDL affects the functionality of LAK cells *in vitro*. We show here that LAK cells take up oxidized-desialylated LDL to a significantly greater extent than native LDL over a period of 72 hours. This resulted in a significant downregulation of LAK cell cytotoxicity against K562 cells. In particular, the expression of IFN-γ, CD56, and NKG2D were reduced upon oxidized-desialylated LDL treatment of LAK cells and, conversely, their expression was enhanced with native LDL. It was also observed that as the number of CD56 and NKG2D positive cells decreased upon treatment with oxidized-desialylated LDL, the number of CD3 positive cells increased in proportion. Additionally, only a slight inhibition of LAK cell cytotoxicity was observed with desialylation alone of LDL, and no significant inhibition was observed with oxidation alone of LDL. Thus, this study describes a new role of oxidized-desialylated LDL as an inhibitor of the antitumor functions of LAK cells. These observations have implications for how atherosclerosis processes, namely oxidation and desialylation of LDL, may influence LAK cell antitumor activity.

## Introduction

Among the immunotherapy methods being considered for anticancer therapy, adoptive transfer of lymphokine-activated killer (LAK) cells is one of the biotherapy approaches that has been translated into clinical studies 1-5. *In vitro* culture of peripheral blood mononuclear cells (PBMCs) with interleukin-2 (IL-2) for several days results in a population of LAK cells with upregulated cytotoxicity against cancer cells and IFN-γ production 6. LAK cells are composed mostly of NK cells (CD3^-^CD56^+^) and NKT cells (CD3^+^CD56^+^), and unlike cytotoxic T cells, LAK cells do not require the major histocompatibility complex (MHC) pathway to perform their antitumor activities 7. This feature makes LAK cells an important anticancer biotherapy for those cancer cells that lack MHC and can't be targeted by T cells. The pathway through which LAK cells target and kill transformed cells is through the Natural Killer Group 2D (NKG2D) receptor 8. The NKG2D receptor is expressed on both NK cells and NKT cells, and it acts as an activation signal when it recognizes its ligands on cancer cells 9. When NK cells and NKT cells are activated through the NKG2D receptor, they secrete apoptosis inducing effectors that kill the cancer cells. Ligands for NKG2D include MICA, ULBP4, and ULBP1, which are implicated in NKG2D mediated recognition of multiple cancers 8, 9.

Several clinical trials have successfully activated and expanded LAK cells using IL-2 1, 2, 7, 10-12. When LAK cells were first introduced by Rosenberg et al in 1985 10, 11, eleven of 25 patients showed significant cancer reduction, with one showing complete tumor regression. Recent studies have shown that the effectiveness of IL-2/LAK immunotherapy can be improved with locoregional administration, rather than by systemic administration of LAKs 12. However, the cytotoxicity activity of LAK cells is ephemeral after they are administered to patients. Studies have shown that LAK cell immunotherapy is more effective with the continuous administration of IL-2 13. After LAK cell administration to patients, LAK cells need to be continuously activated to work. IL-2 activates LAK cells, but it can also inhibit them by inducing the expansion of regulatory T cells 14. Moreover, administration of IL-2 can cause adverse effects on patients, such as vascular leak syndrome 15. Additionally, there are inhibitory factors that inhibit the antitumor functions of LAK cells such as MHC 16, and sialic acid overexpression on cancer cells 17, 18.

Although there are many factors that might be involved in LAK cell inhibition, studies suggest that low-density lipoproteins, free cholesterols, and lipids, can also affect lymphocyte antitumor functions 19-23: Low-density lipoprotein uptake inhibits the antitumor functions of γδT cells, which express NK receptors that determine their antitumor cytotoxicity 19. Inhibition of cholesterol esterification increases CD8^+^ T cell cytotoxic functions 20. NK cells with increased intracellular lipid accumulation display decreased cytotoxicity effects 21, 22. Oxidation of low-density lipoprotein by polymorphonuclear leukocytes inhibits NK activity 23. Oxidation and desialylation of LDL are two important features in the development of atherosclerosis that appear to facilitate the increase of LDL uptake by macrophages 24, 25, smooth muscle cells 26, fibroblasts 27, and aorta cells 28. *In vitro* studies have shown that oxidized-desialylated LDL samples induce an increase in intracellular accumulation of triglycerides, free cholesterol, and cholesterol esters in these cells, as compared to native LDL samples 28. *In vivo* studies suggest that desialylation of LDL appears to be an early event that leads to smaller, denser, more electronegative, and oxidized LDL particles, all of which are referred to as multiple modified LDL, which appear to be a hallmark of atherosclerosis 29, 30. Although the link between multiple modified LDL and cancer immunity remains unclear, several studies have suggested that increased lipid accumulation and increased LDL uptake lead to the impairment of lymphocyte cytotoxic functions against cancer cells 31. However, to our knowledge, there is still no evidence on whether multiple modified LDL affects LAK cell antitumor activity.

In this study, we asked whether the oxidized and/or desialylated forms of LDL modulate LAK cell cytotoxicity towards leukemia cells (K562 cells). We found that LAK cells take up oxidized-desialylated LDL to a greater extent than native LDL, and it causes them to be less cytotoxic against cancer cells. Moreover, oxidized-desialylated LDL uptake drives the downregulation of cytotoxicity associated proteins CD56 and NKG2D, as well as impairment of IFNγ production.

Oxidized-desialylated LDL becomes abundant under the conditions that foster atherosclerosis. Atherosclerosis shares multiple pathways with cancer, and it has been suggested that atherosclerosis promotes tumor development 32. Here we found that oxidation and desialylation are two important posttranslational modifications on LDL that make it an important inhibitor of LAK cell antitumor activity. Thus the results presented here provide a link between factors involved in atherosclerosis and the progression of cancer.

## Results

### Oxidized-desialylated LDL inhibits LAK cell cytotoxicity *in vitro*

**Figure 1:**
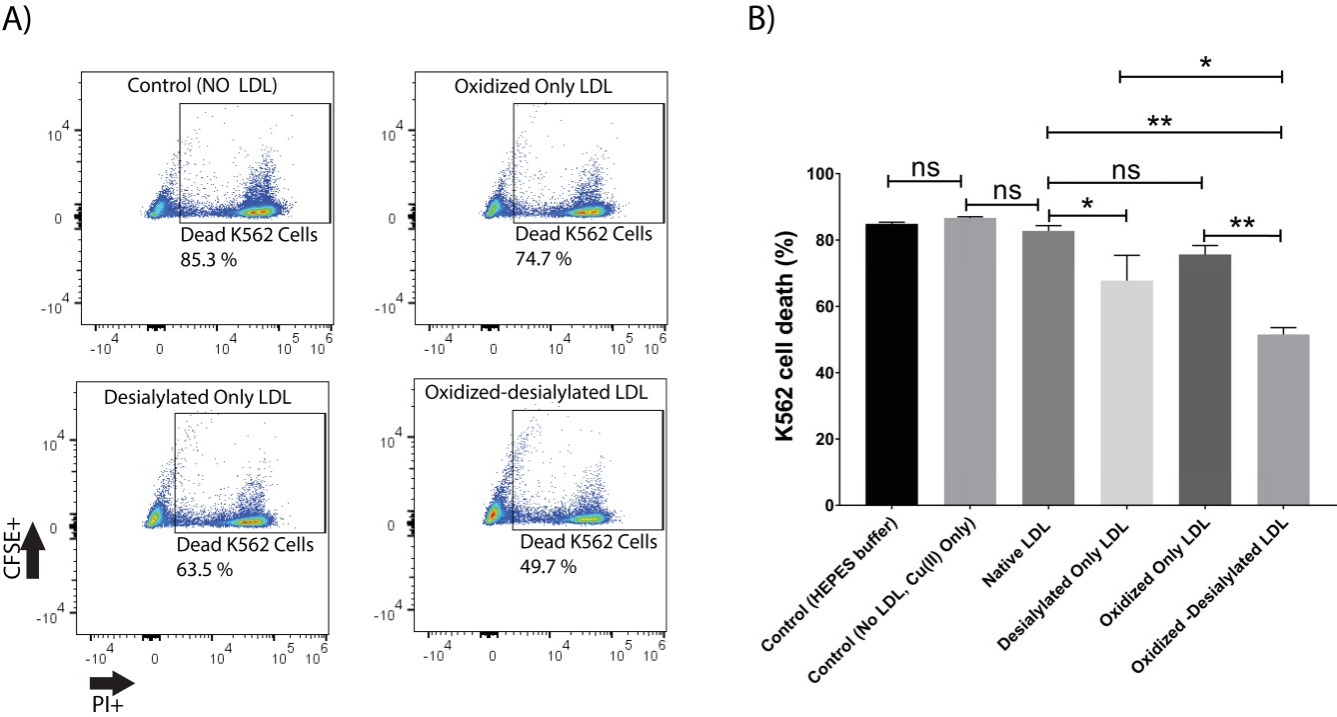
** Oxidized-desialylated LDL inhibits LAK cell cytotoxicity *in vitro*. A)** Activated and expanded LAK cells were cultured in serum free X-VIVO 10 media in a 24 well plate with 0.1 µg/ml IL-2 in the absence or presence of native LDL, oxidized only LDL, desialylated only LDL, or oxidized-desialylated LDL at 50 µg/ml for 72 hours. Then LAK cells were washed three times with X-VIVO 10 serum free media to remove residual external LDL and incubated in a 4-hr killing assay with K562 cells at a 10:1 effector to target ratio. Percent cytotoxicity was determined by flow cytometry. **B)** Quantification of K562 cell death. **indicates a statically significant difference between native LDL (control) and oxidized-desialylated LDL treated LAK cells, and oxidized only LDL vs oxidized-desialylated LDL treated LAK cells (p<0.0001).*Indicates significant difference between native LDL and desialylated only LDL, and desialylated only LDL vs oxidized-desialylated (p <0.001) treated LAK cells, n = 5 per group. ns indicates not statistically significant. Statistical significance determined using two-way ANOVA with Tukey posthoc test. Error bars represent standard deviation.

This study was initiated by investigating the capacity of desialylated LDL to inhibit LAK cell cytotoxicity. For this purpose, PBMCs were activated and expanded *in vitro* for 8 days in the presence of IL-2 to generate lymphokine activated killer (LAK) cells. LAK cells are composed of NK cells (CD3^-^CD56^+^) and NKT cells (CD3^+^CD56^+^). Analysis of IL-2 treated PBMCs by flow cytometry revealed an induced population of cells of which about 6% were NK cells and another 4% were NKT cells (**Supplementary Fig. S1**). This population of cells, referred to as LAK cells, were optimally cytotoxic against K562 cells in a 4-hour killing assay at a 10:1 effector to target ratio (**Supplementary Fig. S2**). Native LDL was desialylated with neuraminidase enzyme and added to the LAK cells at day 5 at 50 μg/ml and incubated for 72 hours. Our glycan node analysis procedure 33-38 was used to verify LDL desialylation prior to adding the LDL samples to the LAK cells. An increase in the relative abundance of terminal galactose, a decrease in 3-linked galactose, and a near-complete loss of 6-linked galactose revealed a major decrease in the number of terminal sialic acid residues present on LDL (**Supplementary Fig. S3**). Only a small inhibition of LAK cell cytotoxicity was observed every time we performed a K562 killing assay after having exposed the LAK cells to desialylated LDL (**Figure 1**). Because previous research has demonstrated that desialylation of LDL appears to be an early event that leads to its oxidation in atherosclerosis 39, we induced oxidation of desialylated LDL with Cu(II) ions. A thiobarbituric acid reactive substances (TBARS) assay revealed that Cu(II)-treated native and desialylated LDL were approximately 50% more oxidized than their untreated LDL counterparts (**Supplementary Fig. S4**). Desialylated and oxidized LDL was then added to the LAK cells at 50 μg/ml (5 mg/dL) and incubated for 72 hours to see how LDL with both modifications affected the cytotoxicity of LAK cells. Significant inhibition of LAK cell cytotoxicity was observed when LDL was both oxidized and desialylated (**Figure 1**). A lesser degree of inhibition was observed when LAK cells were treated with merely desialylated LDL. Oxidation alone did not induce a significant inhibition of LAK cell cytotoxicity (**Figure 1**). To ensure that inhibited K562 cell killing was not simply due to loss of LAK cells, LAK cell viability was checked by incubating the LAK cells with oxidized-desialylated LDL for 72 hours and labeling with Sytox Blue. Cell viability was determined using flow cytometry. Results revealed that treatment of LAK cells with oxidized-desialylated LDL induced a small toxic effect (about 4%) (**Supplementary Fig. S5**). By comparison, the magnitude of the inhibition effect observed in **Figure 1** suggests that the inhibition of K562 cell killing by pre-treating LAK cells with oxidized-desialylated LDL is not simply due to the toxicity of oxidized-desialylated LDL toward LAK cells.

### Modality of K562 Cell Death

**Figure 2:**
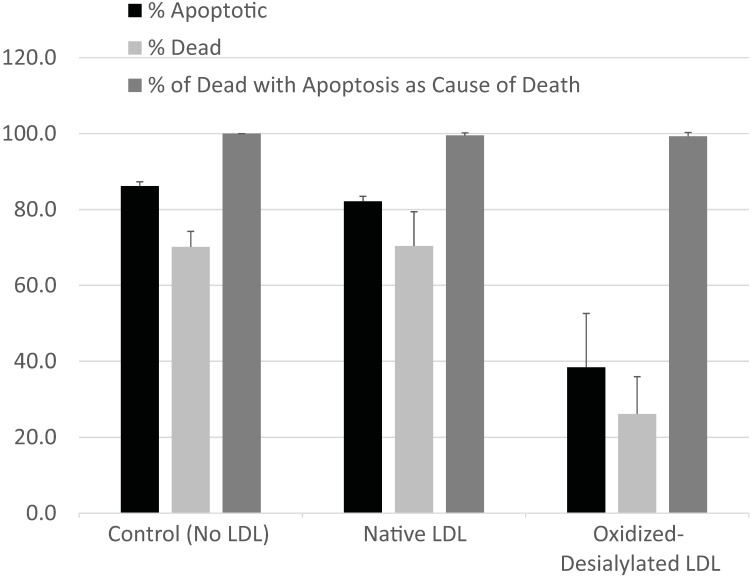
** LAK cells kill target cells via apoptosis.** Target cells (K562 cells pre-labeled with CellTrace Violet) and effector (LAK) cells were incubated together for 4 hrs in the presence of CE dye (apoptosis indicator) and PI (cell death indicator). Percentage of apoptotic K562 cells was determined as 100 x [# of CellTrace & CE-labeled cells] / [# of CellTrace labeled cells]). The percentage of dead K56 was determined as 100 x [# of CellTrace & PI-labeled cells] / [# of CellTrace-labeled cells]). The percentages of K562 that died via apoptosis was determined as 100 x [# of CellTrace & CE & PI-labeled cells] / [# of CellTrace & PI-labeled cells]). A minimum of 130 K562 cells were analyzed in each field. Error bars represent standard deviation from two fields of a representative experiment. No significant differences in the percentage of cells that had died due to apoptosis were detected between LDL treatments (Kruskal-Wallis test).

The type of cell death experienced by K562 cells in the presence of LAK cells exposed to various forms of LDL was evaluated by fluorescence microscopy using CellEvent Caspase-3/7 fluorogenic substrate (CE) which is specific for caspase-3/7 activity and indicative of apoptosis, in combination with CellTrace Violet, a cytoplasmic stain which was used to label K562 cells, and propidium iodide (PI) to identify dead cells. Cells and modified LDL were prepared as described in Materials and Methods. K562 cells, pre-labeled with CellTrace Violet, were incubated with a 10-fold excess of LAK cells that had been exposed to native LDL, oxidized-desialylated LDL, or no LDL at all (control) in assay medium containing CE indicator and PI. In a parallel set of control experiments, the same set of incubations were conducted in the absence of CellTrace Violet and CE dye in order to ensure that neither label altered total cell death. Neither CellTrace nor CE induced cell death of any kind (data not shown). After 4 hrs, samples were imaged by fluorescence microscopy. Images were analyzed to determine the percentage of K562 cells that **1)** were experiencing apoptosis (determined as 100 × [# of CellTrace Violet & CE-labeled cells] / [# of CellTrace Violet-labeled K562 cells]), **2)** had died (determined as 100 × [# of CellTrace Violet & PI-labeled cells] / [# of CellTrace Violet-labeled K562 cells]), or **3)** had died via apoptosis (determined as 100 × [# of CellTrace Violet & CE & PI-labeled cells] / [# of CellTrace Violet & PI-labeled cells]) (**Figure 2**). A minimum of 130 K562 cells were analyzed in each image. Essentially all K562 cell death was due to apoptosis. Moreover, while oxidized-desialylated LDL inhibited K562 cell killing as expected, the type of cell death remained at 99-100% apoptosis regardless of whether the LAK cells were exposed to native LDL, oxidized-desialylated LDL or no LDL at all. Control incubations of K562 cells in the absence of LAK cells revealed < 1% total cell death.

### Enhanced Uptake of Oxidized-Desialylated LDL by LAK Cells

**Figure 3:**
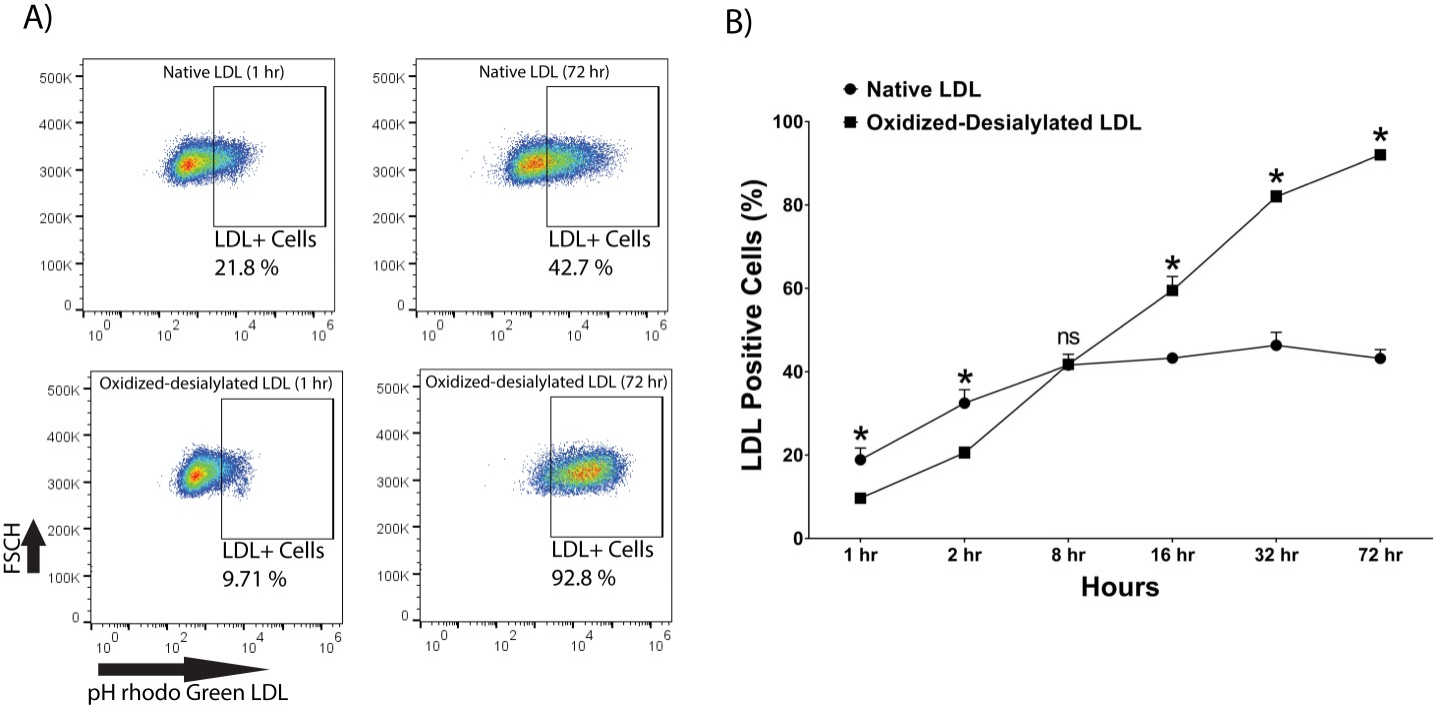
** Enhanced uptake of oxidized-desialylated LDL by LAK cells.** LAK cells were cultured in serum free X-VIVO 10 media in a V-bottom 96 well plate with IL-2 in the absence or presence of native pHrodo Green Conjugate LDL or oxidized-desialylated pHrodo Green Conjugate LDL at 10 µg/ml for 1, 2, 8, 16, 32, and 72 hours. The percentage of LDL positive cells was measured by flow cytometry. **A)** Qualitative flow data showing uptake of oxidized-desialylated LDL and native LDL, which shows the differences in uptake between the two forms of LDL. FSCH stands for forward scatter cell signal height which facilitates selection of single cells. **B)** Time course of native LDL and oxidized-desialylated LDL uptake by LAK cells. * Indicates statically significant differences between native LDL (control) and oxidized-desialylated LDL treated LAK cells (p<0.001). Statistical significance determined using multiple t-tests (one per group) and corrected for multiple comparisons using the Holm-Sidak method. n = 3 per time point. Error bars represent standard deviation. For some points, errors bars are shorter than the symbol, and error bars are not shown.

pHrodo Green-labeled LDL was desialylated with neuraminidase enzyme and oxidized with 10 μM Cu(II) ions. LAK cells were then incubated with native and oxidized-desialylated forms of pHrodo green LDL (**Figure 3**). During the first two hours of incubation, LAK cells took up native LDL faster than oxidized-desialylated LDL. After 8 hours of incubation, the uptake of native and oxidized-desialylated LDL was the same, with no significant difference. However, after 16, 32, and 72 hours, oxidized-desialylated LDL was taken up to a greater extent than native LDL in a time-dependent manner. After 72 hours, a clear increase in oxidized-desialylated LDL uptake was observed (**Figure 3B**). Non-significant differences in uptake were observed for oxidized LDL and, separately, desialylated LDL compared to native LDL (data not shown).

### Oxidized-Desialylated LDL downregulates the cytotoxicity receptors CD56 and NKG2D

**Figure 4:**
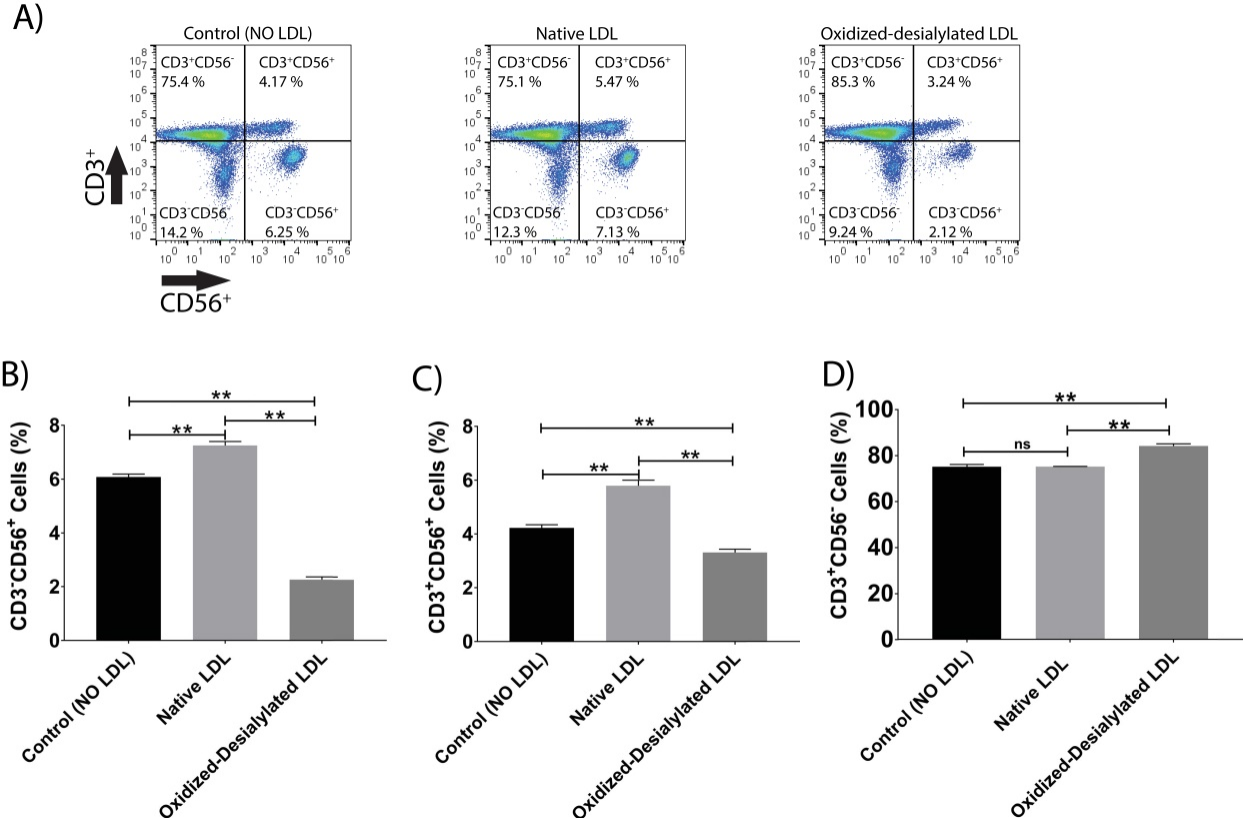
** Oxidized-desialylated LDL downregulates cytotoxicity receptor CD56 and upregulates the CD3 receptor.** Activated and expanded LAK cells were cultured in serum free X-VIVO 10 media in a V-bottom 96 well plate with IL-2 in the absence or presence of native LDL or oxidized-desialylated LDL at 50 µg/ml for 72 hours. Then LAK cells were washed three times with X-VIVO 10 serum free media, reconstituted in PBS, and labeled with anti-CD56 antibodies, as well as with the dead staining dye SytoxBlue.** A)** Live cells were gated and plotted against CD3 and CD56. **B)** Oxidized-desialylated LDL decreased the number of CD3^-^CD56^+^ cells. **C)** The number of NKT cells (CD3^+^ CD56^+^) also decreased significantly. **D)** The number of CD3 positive cells increased significantly upon oxidized-desialylated LDL treatment of LAK cells. n = 5 per group. Error bars represent standard deviation. ** indicates statically significant differences (p<0.0001), determined using one-way ANOVA with Tukey posthoc test.

LAK cells express the surface markers CD56, CD3, and NKG2D. CD56 and NKG2D are cytotoxicity associated receptors expressed on both NK cells and NKT cells, and CD3 is an activating receptor expressed on NKT cells and T cells 40. Because LAK cell anti-cancer (anti-K562) activity was reduced in the 4-hour killing assay upon LAK cell uptake of oxidized-desialylated LDL over a 72-hour period, we tested whether the expression levels of these receptors were also affected. After gating all the live cells and plotting them against CD3 and NKG2D, a decrease in the proportion of CD56^+^ cells and CD3^+^CD56^+^ cells was observed (**Figure 4A-C**). Interestingly, it was observed that as the number of CD3^-^CD56^+^ cells and CD3^+^CD56^+^ cells decreased, the number of CD3^+^-only cells increased (**Figure 4D**). This inverse relationship implies that oxidized-desialylated LDL causes LAK cells to differentiate into CD3^+^-only rather than CD56^+^ cells, making them less cytotoxic against K562 cancer cells. It was also observed that native LDL induced a small increase in the number of CD56^+^ cells, although in the 4-hour killing assay no change in cytotoxicity was observed.

**Figure 5:**
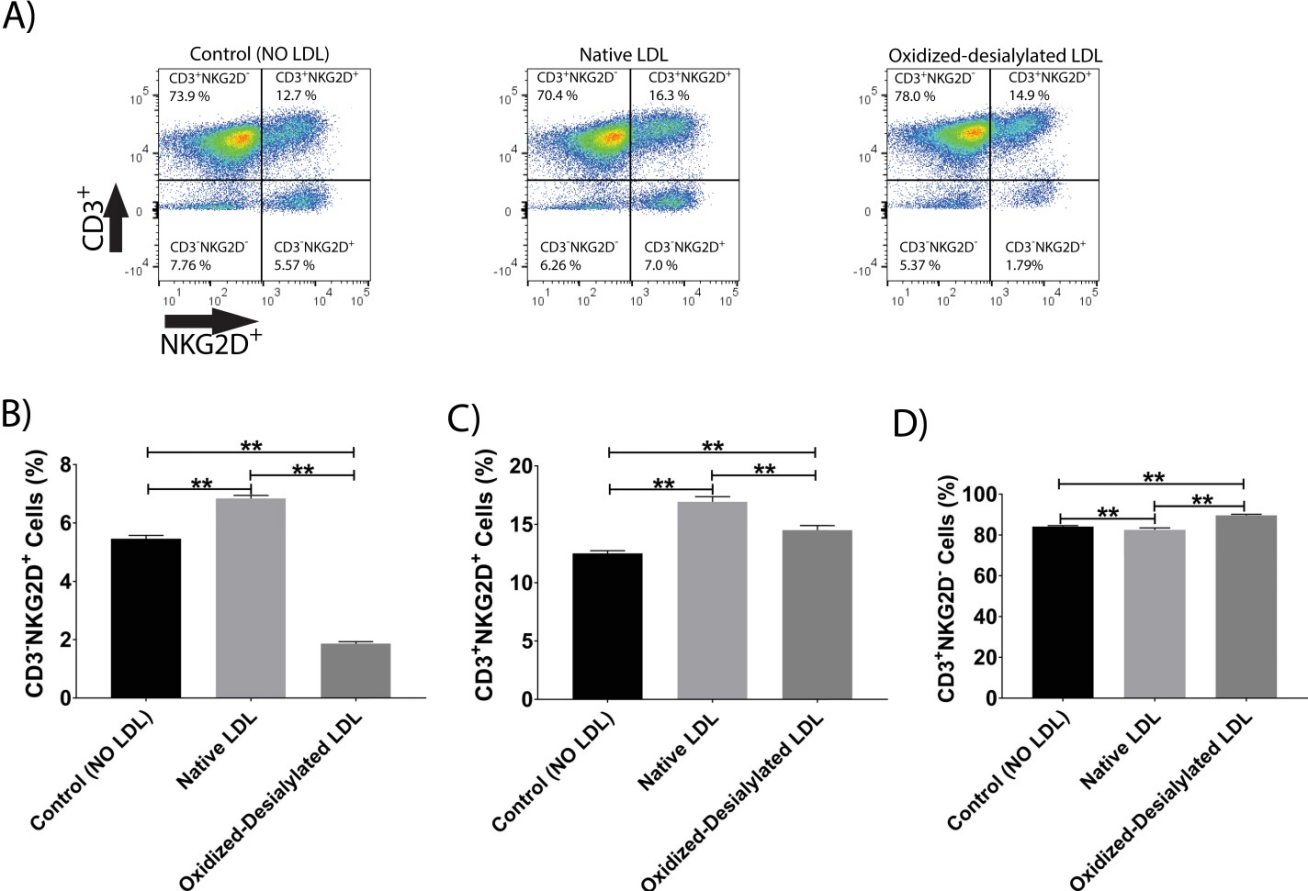
** Oxidized-desialylated LDL downregulates activating receptor NKG2D**. LAK cells were cultured and washed as described in Fig. 3. Then LAK cells were labeled with anti-NKG2D antibodies, as well as with the dead staining dye SytoxBlue. **A)** Live cells were gated and plotted against CD3 and NKG2D. **B)** Oxidized-desialylated LDL decreased the number of CD3^-^NKG2D^+^ cells. **C)** Oxidized-desialylated LDL increased the number of CD3^+^ cells relative to the LDL-free control, but decreased them relative to treatment with native LDL. **D)** Oxidized-desialylated LDL increased the percentage of CD3^+^NKG2D^-^ cells relative to both other groups. n = 5 per group. **Indicates statistically significant differences (p<0.0001), determined using one-way ANOVA with Tukey posthoc test. Error bars represent standard deviation.

Additionally, it was observed that native LDL induced a small increase in the expression of NKG2D receptor (**Figure 5A-C**). However, oxidized-desialylated LDL downregulated the percentage of CD3^-^NKG2D^+^ cells while slightly increasing the percentage of CD3^+^NKG2D^+^ cells (**Figure 5A-C**). A small increase in the number of CD3^+^ only cells was also observed in the oxidized-desialylated treated samples (**Figure 5D**).

### Oxidized-desialylated LDL impairs Interferon Gamma (IFN-γ) production

**Figure 6:**
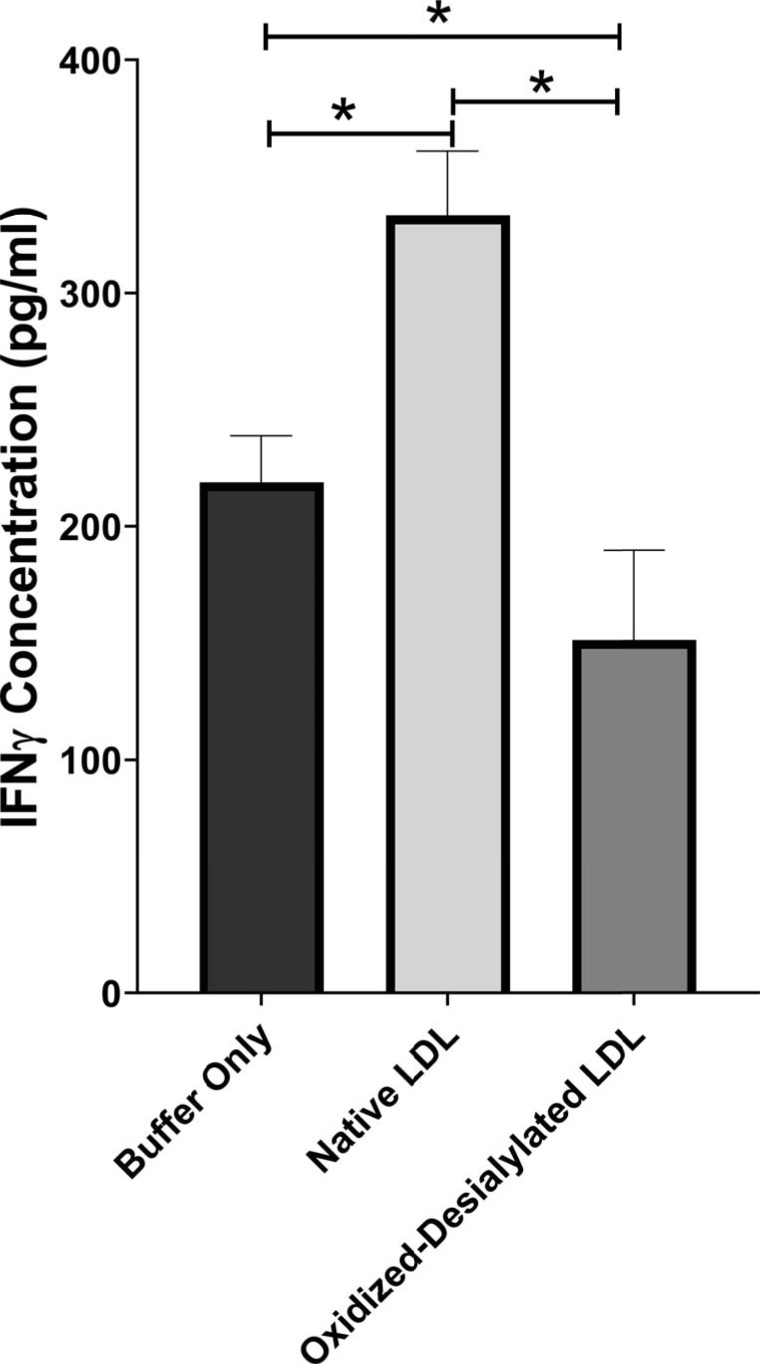
** Oxidized-desialylated LDL impairs IFNγ production.** LAK cells were cultured in serum free X-VIVO 10 media with 0.1 µg/ml IL-2 in the presence or absence of native LDL or oxidized-desialylated LDL for 72 hours. After 72 hours, cell supernatants were removed, centrifuged at 330 g for 8 minutes. Soluble IFNγ was measured in undiluted cell supernatants by ELISA. *Indicates statically significant differences between native LDL (control) and oxidized-desialylated LDL treated LAK cells (p<0.001). Statistical significance determined using one-way ANOVA with Turkey posthoc test. n = 4 per group. Error bars represent standard deviation.

Interferon gamma (IFN-γ) is a pleiotropic cytokine with antitumor functions. IFN-γ is secreted by NK cells and NKT cells, and it is often considered the major effector of immunity 41. Because oxidized-desialylated LDL caused an inhibition of LAK cell cytotoxicity and downregulation of the cytotoxicity receptors, we investigated whether production of IFN-γ was altered during the process. An ELISA was performed to quantitively measure soluble IFN-γ in LAK cell supernatants that had been treated with native LDL or oxidized-desialylated LDL. Results showed that oxidized-desialylated LDL decreased the production of IFN-γ. Previous research has shown that native LDL downregulates the production of IFN-γ in γδT cells 19 - prompting expectations of a similar result. Unexpectedly, however, native LDL significantly enhanced the production of IFN-γ in LAK cells (**Figure 6**).

## Discussion

Cancer and atherosclerosis are distinct diseases that share several molecular pathways, with some studies suggesting that atherosclerosis can lead to cancer development 32. Characteristic features of both diseases include inflammation, uncontrolled cell proliferation, and oxidative stress 42. Atherosclerosis is characterized by chronic inflammation, diabetes, hypertension, dyslipidemia, and obesity, all of which are predisposing risk factors for cancer 42. At the molecular level, inflammation leads to the production of inflammatory cytokines, such as IL-6, IL-1β, and TNF, which have been associated with cardiovascular disease and several different types of cancer 43. High concentrations of biomarkers for reactive oxygen species such as oxidized LDL and myeloperoxidase (MPO), have been found in the blood plasma of cancer patients and atherosclerosis patients, and the origin of both diseases is associated with oxidative stress 44, 45. Additionally, it has been found that the metabolism of unstable atherosclerotic plaques is similar to that of cancer cells 46.

Oxidation and desialylation of low-density lipoprotein are hallmark modifications of LDL in atherosclerosis that contribute to the initiation and progression of the disease 29. These modifications are believed to be needed to facilitate increased LDL uptake by atherosclerotic cells and cause them to have increased intracellular triglycerides, free cholesterol, and cholesterol esters 29, 30. Although little is known about oxidized-desialylated LDL in cancer, literature suggests that oxidized LDL is involved in tumor development 47. Long term exposure of human vascular smooth muscle cells (hVSMC) to chemically oxidized LDL promotes the overexpression of osteopontin, a glycoprotein involved in cancer metastasis 48. Inflammation is a characteristic feature of both atherosclerosis and cancer, and studies have shown that oxidized LDL triggers inflammation. Macrophages take up oxidized LDL via scavenger receptors which, once inside the cell, induce cholesterol crystals that ultimately activate the inflammasome to release IL-1β and TNF cytokines, which are mediators of inflammation in both cancer and atherosclerosis 49-52.

Although it has been suggested that oxidized-LDL plays a role in cancer development 53, little is known about the role that oxidized and desialylated LDL plays in antitumor immunity. It has been previously shown that accumulation of native LDL in γδ T cells leads to reduced antitumor function 19. A different study showed that when LDL becomes oxidized by polymorphonuclear lymphocytes, it inhibits NK cell cytotoxicity 23. However, to our knowledge the role of LDL that is both oxidized and desialylated on the cytotoxicity of LAK cells has been unknown until now. Oxidation and desialylation are characteristic features of LDL in atherosclerosis 29; the meaning of this in cancer immunity is still not well understood. We found that oxidized-desialylated LDL is taken up by LAK cells more extensively than native LDL after 16 hours of incubation, which leads to reduction of LAK cell cytotoxicity. This reduction in cytotoxicity was not, however, accompanied by a shift in the mechanism of K562 cell death; it was fully apoptotic under all experimental conditions tested (**Figure 2**). This observation was in line with previous studies on the mechanism by which LAK cells kill cancer cells 54-56.

The data presented here showed that the expression of the cytotoxicity-associated receptors CD56 and NKG2D, which determine the ability of LAK cells to mediate cancer cell lysis 57, was downregulated upon exposure of LAK cells to oxidized-desialylated LDL. This occurred in pre-expanded LAK cells that were kept on IL-2 stimulation during the incubation with oxidized-desialylated LDL. Thus IL-2 stimulation was not able to compensate for the inhibitory effect of oxidized-desialylated LDL. We also found an impairment in the production of IFNγ, a pleiotropic cytokine secreted by LAK cells. These results, however, differ according to the use of different cytotoxic lymphocytes. For instance, a different study found that native LDL can lower the expression of CD56 and NKG2D cytotoxicity receptors and IFN-γ in γδT cells 19. However, we observed the opposite in that native LDL induced a small increase in the expression of CD56, NKG2D, and IFN-γ in LAK cells. Nevertheless, previous studies and our work show that enhanced uptake of LDL by cytotoxic immune cells can impair their antitumor capacity.

Multiple modified LDL has been extensively studied in the context of atherosclerosis, and how it can contribute to the development of this disease 58. Although it is unknown how LDL becomes multiply modified in atherosclerosis, our findings suggest that the desialylated and oxidized LDL that is produced as a byproduct of atherosclerosis may at least partially inhibit the ability of the immune system to effectively surveil against cancer cells. Thus, the data presented here suggests at least one particular mechanistic link between atherosclerosis and cancer. Future research investigating the link between multiple modified LDL and cancer immunity will help establish additional details on the complex connection between these two devastating chronic diseases.

## Materials and Methods

### Cell culture and *in vitro* cytotoxicity assay

For LAK cell culture and expansion, peripheral blood mononuclear cells (PBMCs) were isolated by density gradient centrifugation (Ficoll-Paque-GHC-17-1440-02; GE Healthcare) from Trima Residual Apheresis collection kits (RE202-Blood Centers of the Pacific). Trima residuals were centrifuged at 1200 g for 20 minutes at 15 °C. Isolated PBMCs were cultured in serum free X-VIVO 10 medium with Gentamicin L-Gln and Phenol Red (04-380Q; Lonza) for 5 days in the presence of interleukin-2 (IL-2, 589102; BioLegend) at 0.1 μg/ml. At day 5, fresh media was added along with fresh IL-2, and LAK cell populations were cultured for 72 hours in the presence or absence of native low-density lipoprotein (LDL, 12-16-120412; 50 μg/ml, Athens Research & Technology) or oxidized-desialylated low-density lipoprotein, and LAK cell cytotoxicity was evaluated in an *in vitro* killing assay. Cells were counted via hemocytometer and Trypan blue solution (0.4%). K562 leukemia cells were cultured in RPMI medium supplemented with 10% fetal bovine serum (FBS, SER-500, Zen-Bio). For the *in vitro* killing assays, about 800,000 LAK cells were washed three times in 2 ml serum free X-VIVO 10 media and seeded in a V-bottom 96 well plate. K562 cells were washed two times with 5 ml 1X PBS and stained with CFSE green dye (0.25 μmol/L in 1X PBS; Thermo Scientific) for 10 minutes at room temperature. Eighty microliters of target K562 cells at 10^6^ cells/ml were added to a V-bottom 96 well plate containing 100 μl of the LAK cells at a 10:1 effector to target ratio, and incubated for 4 hours at 37 °C and 5% CO_2_. Immediately afterward cells were centrifuged at 800 × g for 10 minutes and reconstituted in 700 μl of 1X PBS, and to this was added 2 μl of a 1 mg/ml propidium iodide solution (P3566; Thermo Scientific) which was incubated for 20 minutes and subsequently analyzed by flow cytometry.

### Cell Death Analysis

About 2 × 10^6^ K562 cells were incubated with CellTrace Violet (C34571, Thermo Fisher Scientific) at a final working concentration of 20 μM and incubated for 30 minutes. Then, labeled K562 cells were washed once with 5 ml sterile 1X PBS and resuspended in RPMI medium containing 10% FBS, 5 μM CellEvent Caspase-3/7 Green Detection Reagent (C10723, Thermo Fisher Scientific) and 1 μg/ml propidium iodide. LAK cells were incubated with different forms of LDL and prepared as described above, and then they were reconstituted in X-VIVO 10 media containing 5 μM CellEvent and 1 μg/ml propidium iodide. These LAK cells were co-incubated with CellTrace-loaded K562 cells for 4 hours at a 10:1 ratio, as described above. After incubation, samples were imaged on a Nikon Ti-2, inverted fluorescence microscope using a 20 lens with N.A. of 0.75. Each fluorescent channel and differential interference contrast (DIC) images were captured sequentially. Excitation/emission wavelengths for the blue (CellTrace), green (CellEvent) and red (propidium iodide) channels were 405/450, 488/525, and 561/600 nm, respectively. Two random fields were imaged for each sample.

**Figure 7:**
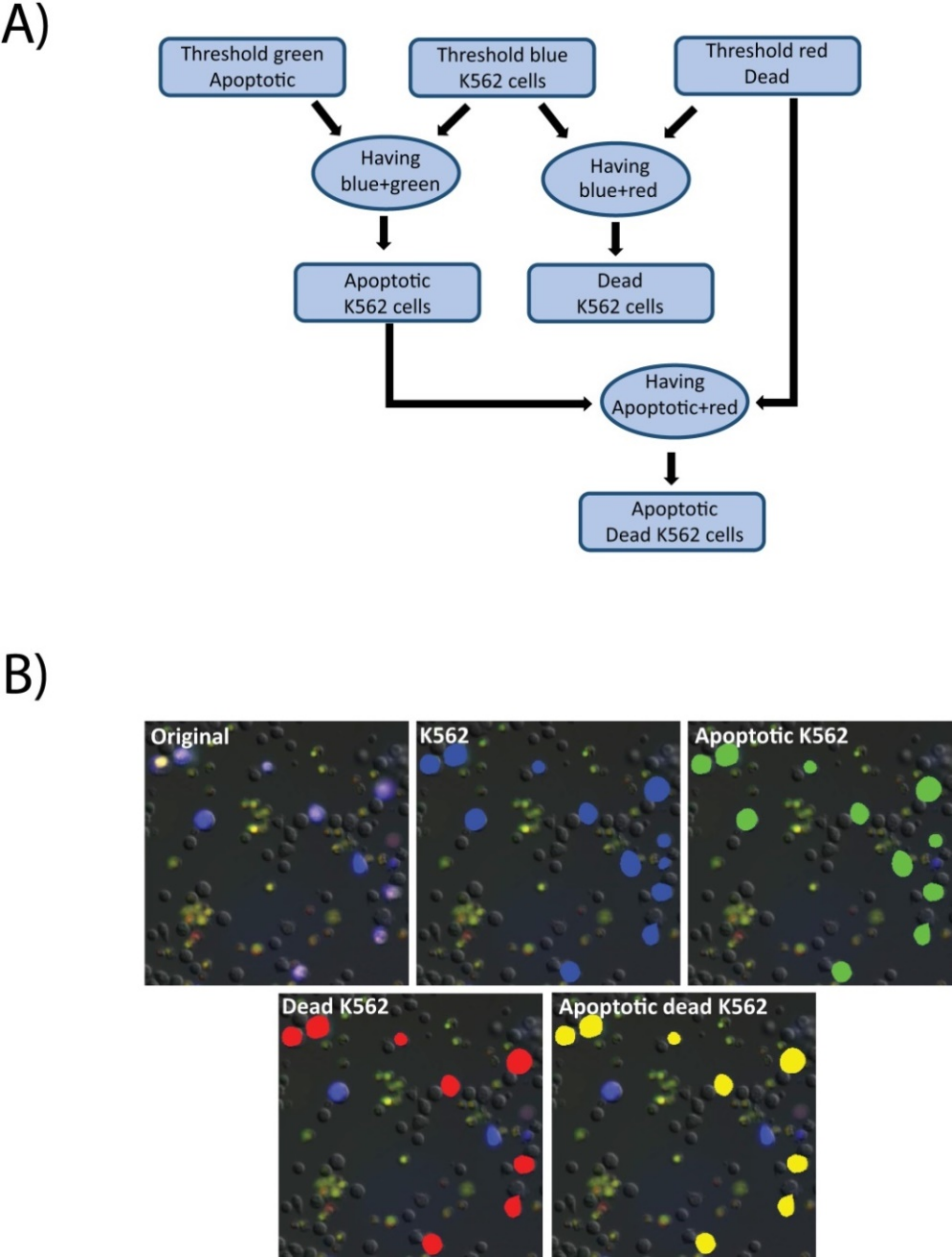
** Cell death image analysis pipeline. A)** A macro was created in the Nikon Elements software for image analysis. First the images were thresholded in the green, blue, and red channels representing, respectively, the apoptotic cells (labeled with CellEvent), the K562 cells (labeled with CellTrace Violet), and the dead cells as defined as loss of membrane integrity (labeled with propidium iodide). The having command was used to identify apoptotic K562 cells and dead K562 cells. Having was then used again to identify K562 cells that were dually positive for apoptosis and death indicators. **B)** Raw images (original) were processed through the pipeline to identify K562 cells (blue mask), apoptotic cells (green mask), dead K562 cells (red mask) and Apoptotic, dead K5562 (yellow mask).

Images were analyzed using the Nikon Elements software. First a threshold was applied to the raw images in the blue channel to detect all K562 cells. Then the green and red channels were thresholded to define positive signal indicating apoptotic and dead cells, respectively. The “having” command was then used to select all detected K562 cells that contained any above-threshold green signal. The results were identified as apoptotic K562 cells. Similarly, having was employed to detect K562 cells with above-threshold red signal, to select dead K562 cells. Finally, having was applied to identified apoptotic K562, and red signal to identify K562 cells that were dead via apoptosis (**Figure 7A**). A cropped field in shown in **Figure 7B** indicating the output of the analysis steps. The initial image is an overlay of the fluorescence and DIC channels (**7B**, original), identified K562 cells are identified by a blue mask (**7B**, K562), the results of K562 cells that also contain the apoptosis indicator are masked in green (**7B**, Apoptotic K562), K562 positive for propidium iodide, indicating loss of membrane integrity are masked in red (**7B**, Dead K562), and the K562 positive for both indicators are indicated in yellow (**7B**, Apoptotic K562).

### Desialylation of low-density lipoprotein and glycan node analysis

*In vitro* desialylation of native low-density lipoprotein (LDL) was performed by incubating 50 μl of a 7.11 mg/ml LDL stock with 100 μl of 0.1M sodium acetate buffer pH 5, plus 50 μl of neuraminidase enzyme from Clostridium perfringens (1 U/ml, 11585886001; SIGMA) or 50 μl of water for the control. Total volume was brought up to 250 μl with water, and samples were incubated at 37 ^0^C for 24 hours. To remove neuraminidase enzyme, samples were spin filtered using Amicon Ultra-0.5 Centrifugal 100 kD spin filter devices (UFC510024; Fisher Scientific). Samples were reconstituted in 450 μl of 10 mM HEPES in 0.15 M NaCl pH 7 buffer each time for a total of 4 spin throughs. To verify complete desialylation of LDL, the glycan node analysis method 33-38 was used. Glycan node analysis is a procedure based on glycan methylation analysis by which pooled glycans within whole biological samples are deconstructed in a way that conserves their monosaccharide and linkage information 33. For this procedure, 60 μg of LDL were aliquoted, and to this was added 270 μl of DMSO and 105 μl of iodomethane. Then samples were added to sodium hydroxide beads for 11 minutes to drive permethylation. The rest of the steps for glycan linkage analysis of intact, complex biospecimens are described elsewhere 33-38. An increase in the relative abundance of terminal galactose and a near-complete loss of 6-linked galactose signals (which arise solely due to terminal sialylation of glycans) were used to verify desialylation of LDL 33.

### Oxidation of desialylated low-density lipoprotein and TBARS assay

A 200 μM copper II chloride solution was prepared using acetic acid pH 4. Desialylated LDL and/or native LDL at 1.7 mg/ml was incubated with 10 μM copper II chloride overnight at 4 °C to generate oxidized-desialylated and/or oxidized only LDL. For the control, native LDL was incubated with a blank acetic acid solution pH 4 at 4 °C overnight. Oxidation was verified with the thiobarbituric acid reactive substances (TBARS) assay, as described by Aguilar Diaz de leon & Borges 59. Briefly, to 100 μl of an LDL sample (60 μg) or calibration standards were added 200 μl of 8.1% sodium dodecyl sulfate, 1.5 ml of 20% acetic acid solution adjusted to pH 4 with NaOH, and 1.5 ml of 0.8 % aqueous solution of thiobarbituric acid. Final volume was brought up to 4 ml with water. The mixture was heated at 95 °C for 1 hour and centrifuged at 1600 g at 4 °C. One hundred fifty microliters of supernatant was transferred to a 96 well plate, and the absorbance was measured immediately at 532 nm.

### Flow-cytometry analysis

For antibody staining, LAK cells and PBMCs were labeled with monoclonal antibodies: anti-CD3, anti-CD56, and APC anti-human CD314 (NKG2D) (1D11, BioLegend). The percentage of CD3, CD56, and NKG2D positive cells was evaluated by flow cytometry in a ThermoFisher Attune NxT flow cytometer and FlowJo software. For LDL uptake measurements, 900,000 LAK cells were seeded in a 96 well plate with 225 μl X-VIVO 10 media and incubated with either native or oxidized-desialylated pHrodo Green LDL (L34355, Thermo Scientific) at 10 μg/ml for 1, 2, 8, 16, 32, and 72 hours. LAK cells were washed three times with 2 ml serum free X-VIVO 10 media, reconstituted in 700 μl 1X PBS, and immediately analyzed by flow cytometry.

### Enzyme Linked Immunosorbent Assay (ELISA) of Interferon Gamma (IFNϒ)

IFNγ was quantitatively measured in undiluted samples of LAK culture supernatant. The wells of a high binding flat bottom 96-well plate (Costar, Washington, D.C.) were first coated overnight at 4 °C with 100 µl of 1 µg/mL solution of α-Human IFNγ monoclonal antibody (Clone 1-D1K, Mabtech, Sweden) diluted in bicarbonate buffer (1 to 100 dilution), pH 9.8. The wells were then washed 5 times with 200 μl PBS-T buffer. Next, 200 µl of 5% milk in PBS-T blocking buffer was incubated in each well for 1 hour at room temperature. Standards were diluted in PBS-T containing 5% powered milk and ranged from 2 ng/mL to 34 pg/mL of recombinant human IFNγ (R&D Systems, Minneapolis, MN). One hundred microliters of cell culture supernatants were plated in triplicate at dilutions of 1:10, 1:100, and 1:500 in 200 μl PBS-T 5% milk and incubated for 2 hours at room temperature. Samples were washed 5 times with 200 μl PBS-T. Biotinylated, secondary α-Human IFNγ monoclonal antibody (Clone 7-B6-1, Mabtech, Sweden) was diluted to 1 µg/mL in PBS-T containing 5% powered milk and 100 µl was incubated in each well overnight at 4 °C. Samples were once again washed 5 times with PBS-T, after which Precision Protein StrepTactin-HRP Conjugate (Bio-Rad, Hercules, CA) was diluted 1:250 in PBS-T containing 5% powered milk and 100 µl incubated in each well for 30 minutes at room temperature. After 5 more washes with PBS-T, 100 µl of TMB substrate (Thermo Scientific, Waltham, MA) was added to each well and allowed to incubate until a visible gradient developed in the standards, which was about 5 minutes. Subsequently, 100 µl of 2N sulfuric acid stop solution was added and the plate was read at 450 nm immediately.

### Statistical analysis

Statistical analysis was performed using two-way ANOVA with multiple comparison test for comparison of experimental groups with cytotoxicity assays, TBARS assays, and flow cytometry analysis. For glycan linkage analysis, each uniquely linked monosaccharide was quantified by integrating extracted ion chromatogram peak using QuanLynx software. Integrated peaks were exported to an Excel spreadsheet. Statistical significance between glycan nodes was determined using two-tailed t-tests. Statistical analysis was performed using GraphPad.

## Supplementary Material

Supplementary figures.
