# Supplementary material for: Oxidized-Desialylated Low-Density Lipoprotein Inhibits the Antitumor Functions of Lymphokine Activated Killer Cells
Source: J Cancer. 2021 Jun 16;12(16):4993–5004. doi: 10.7150/jca.55526 (PMC8247392; doi:10.7150/jca.55526)
Supplement: Supplementary file 1 — Supplementary figures. [file jcav12p4993s1.pdf]

A)

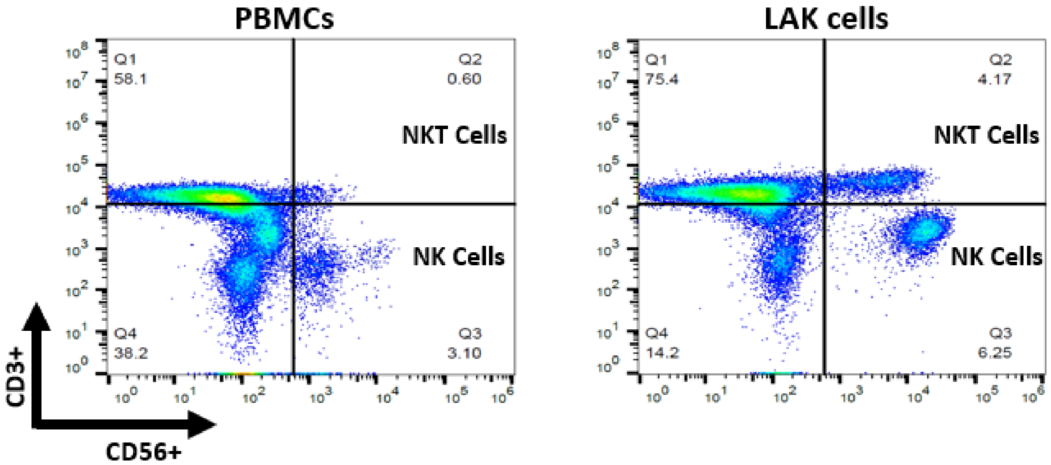

B)

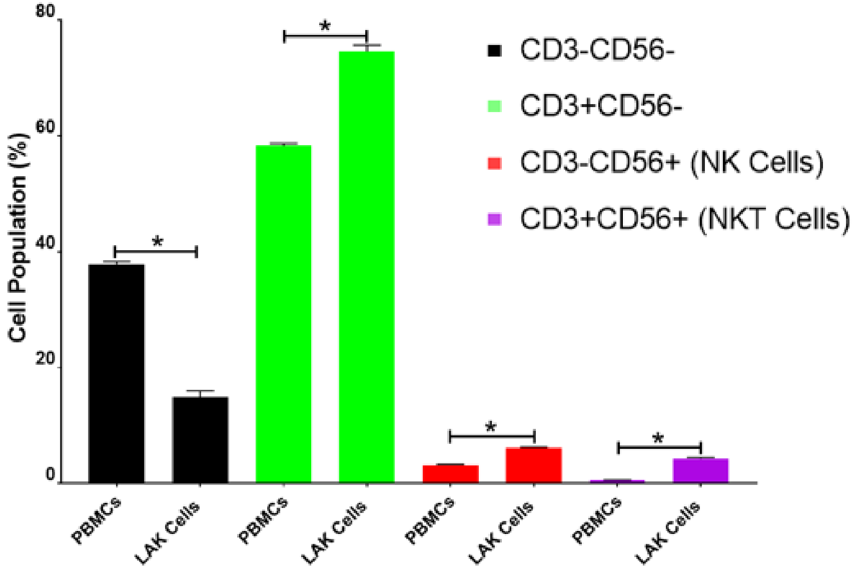

Supplementary Figure 1. LAK cell phenotype. **A)** Lymphokine Activated Killer (LAK) cells (**right**), induced by exposure of PBMCs (**left**) to IL-2 for 8 days are composed of NK cells ($CD3^{-}CD56^{+}$) and NKT cells ($CD3^{+}$ $CD56^{+}$). **B)** CD3 and CD56 marker expression on PBMCs vs. LAK cells. n = 4 per group. * indicates a statistical significance (p<0.001). Error bars represent standard deviation. Statistical significance determined using individual two tailed t-tests.

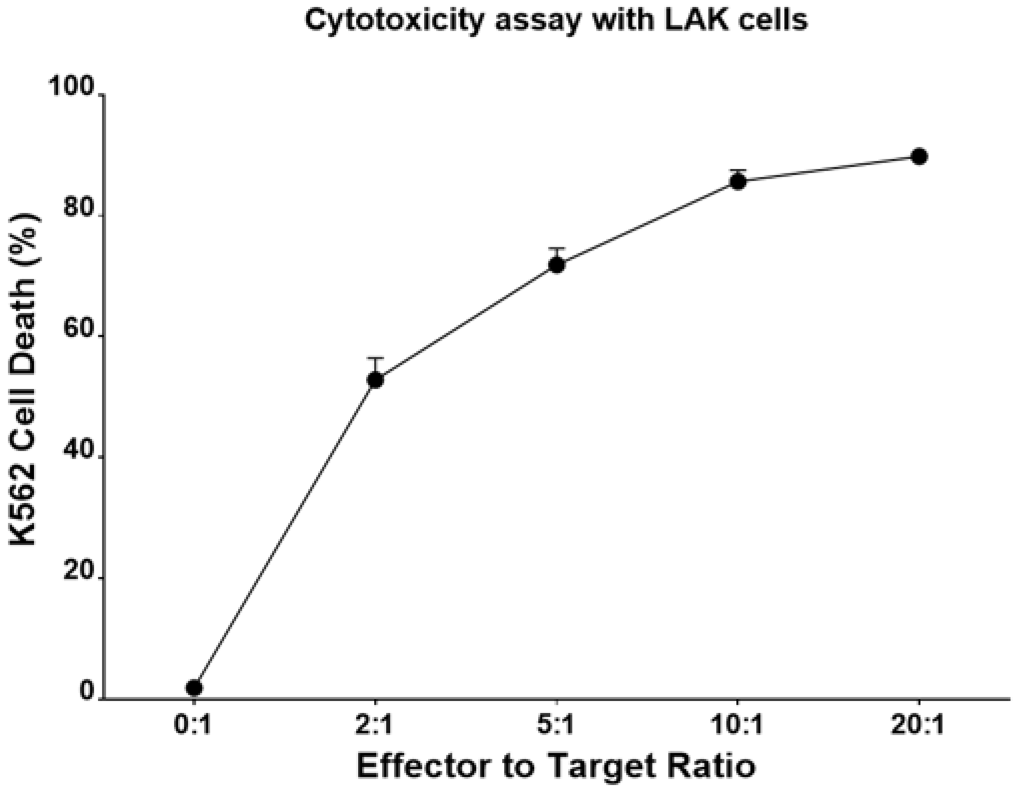

Supplementary Figure 2. Cytotoxicity assay effector cell ratio optimization. Lymphokine Activated Killer (LAK) cells were optimally cytotoxic against K562 cells at a 10:1 effector to target ratio. PBMCs were incubated with 0.1 µg/ml IL-2 for 8 days to generate LAK cells. K562 cells were labeled with CFSE green dye and co-incubated with unlabeled LAK cells for 4 hours at different effector to target ratios. Error bars represent standard deviations. n = 4 per group.

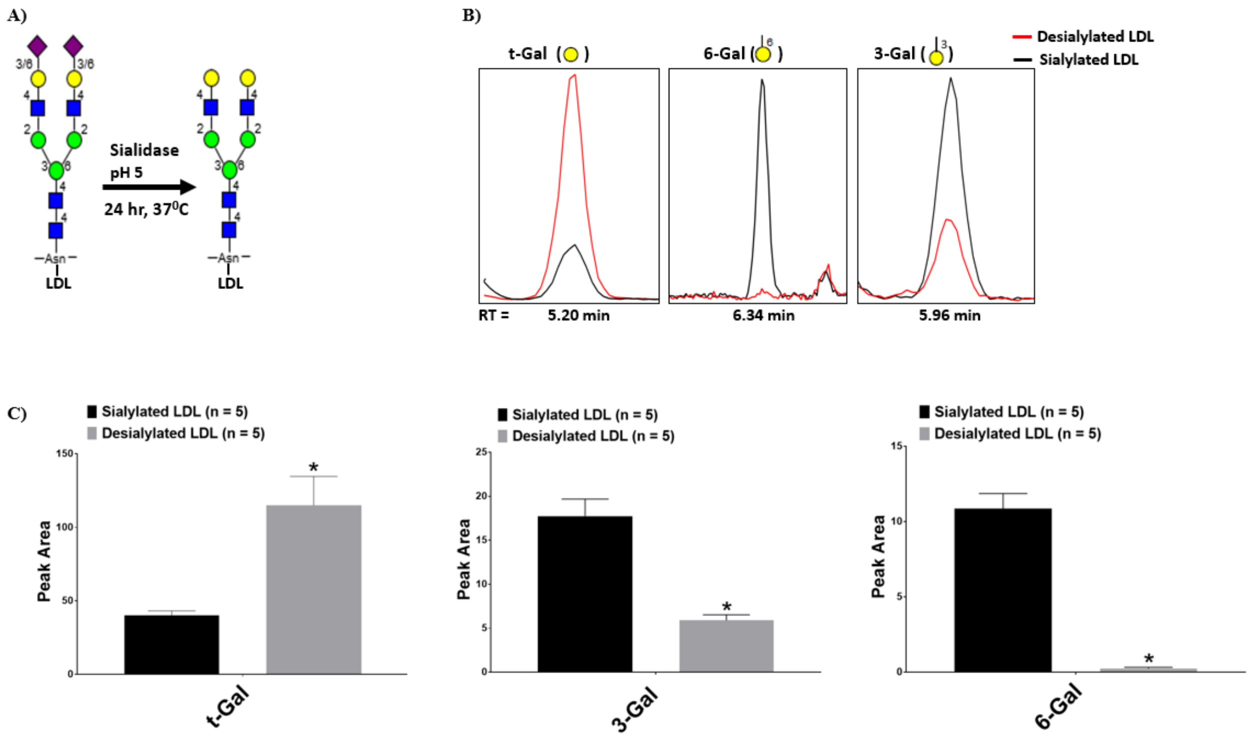

Supplementary Figure 3. ***In vitro* LDL desialylation and glycan node analysis. A)** LDL samples were desialylated with neuraminidase enzyme (1 U/ml) in 0.1 M sodium acetate buffer pH 5 at 37 ^{0}C for 24 hours. Representative molecular changes are illustrated. **B)** Summed extracted ion chromatograms at *m/z* 117.05+ 145.1 for terminal galactose (t-Gal), *m/z* 161.1 + 233.1 + 189.1 for 6-linked galactose (6-Gal), and 161.1 + 233.1 for 3-linked galactose (3-Gal) show a significant decrease in terminal sialylation, as determined by glycan node analysis [33–38]. **C)** Quantitative summary of illustrative data shown in B that reveal a major increase in terminal galactose residues, a decrease in 3-linked galactose residues, and a near-complete loss of 6-linked galactose residues, indicating an overall decrease in terminal neuraminic acid residues. Statistical significance determined using a two tailed t-test. n = 5 per group. * indicates a statistically significant difference ($p<0.001$). Error bars represent standard deviation.

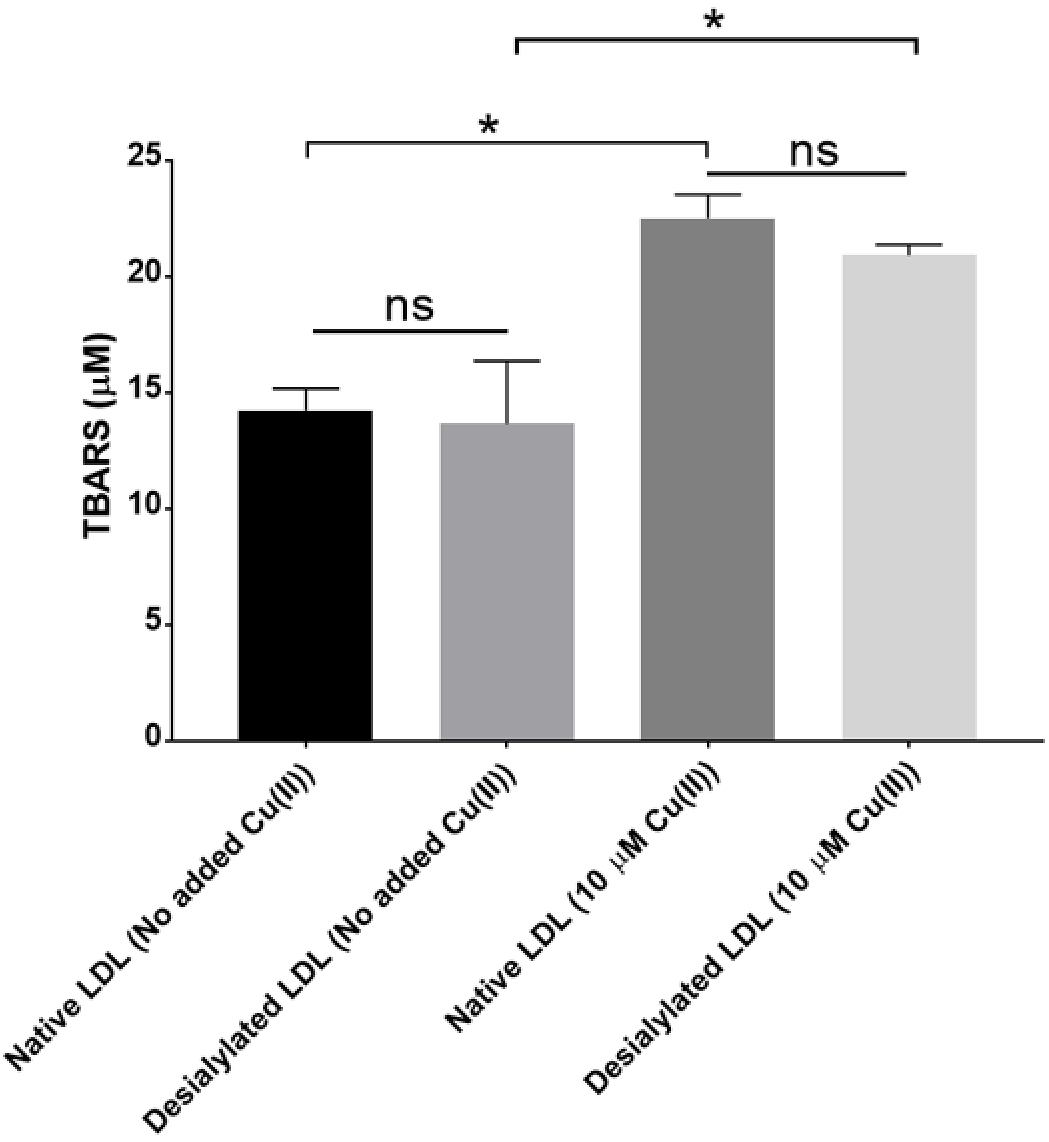

Supplementary Figure 4. ***In vitro LDL oxidation.*** After desialylation, samples were incubated with 10 μM Cu (II) ions for 24 hours at 4 ^{0}C to drive oxidation of LDL. Levels of LDL oxidation were verified by measuring the production of thiobarbituric acid reactive substances (TBARS) [59]. Error bars represent standard deviation. * indicates statistically significant differences between samples ($p<0.001$) and ns indicates no significant difference. n = 5 per group. Statistical significance determined using a two-way ANOVA test with Tukey posthoc test.

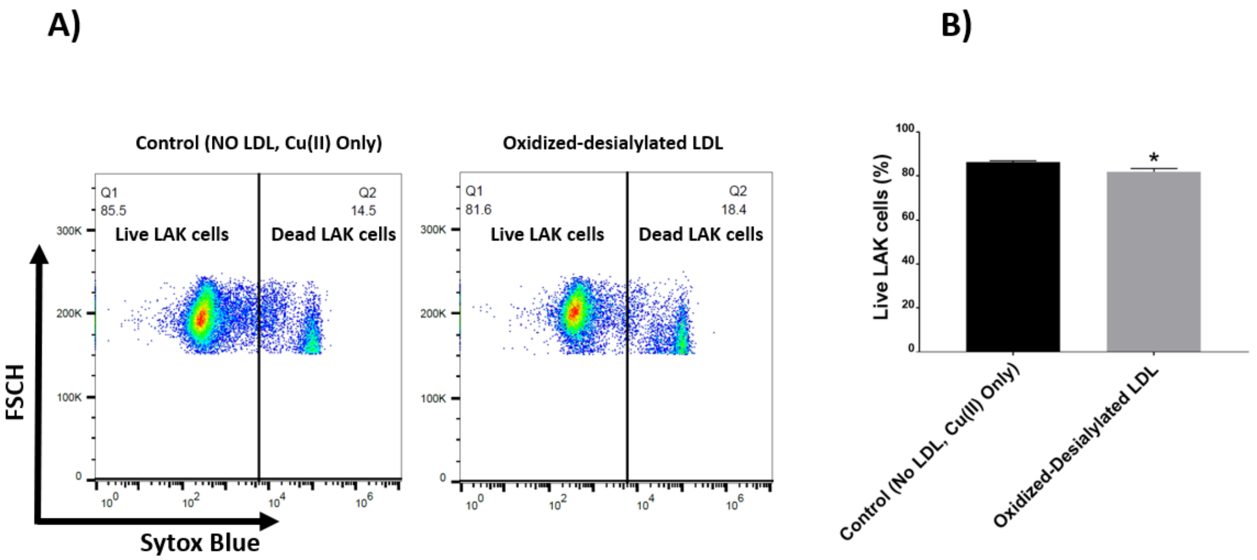

Supplementary Figure 5. LAK cell viability.

A) LAK cells were incubated with oxidized-desialylated LDL for 72, then LAK cells were stained with Sytox blue and analyzed by flow cytometry to determine LAK cell viability. FSCH stands for forward scatter cell signal height which facilitates selection of single cells. **B)** Oxidized-desialylated LDL was slightly toxic to the LAK cells. n = 4 per group. Statistical significance determined using a two-tailed t-test. * indicates a statistically significant difference ($p = 0.028$). Error bars represent standard deviation.
